# Treatment of symptomatic hip dysplasia by posterolateral small incision assisted Bernese periacetabular osteotomy

**DOI:** 10.1186/s12893-022-01666-0

**Published:** 2022-06-06

**Authors:** Chuan Li, Xianghong Zhang, Xuhan Meng, Luqiao Pu, Hongxuan Chen, Yongyue Su, Pengfei Bu, Yongqing Xu, Tang Liu

**Affiliations:** 1 Department of Orthopedics, The 920th Hospital of Joint Logistics Support Force of Chinese People’s Liberation Army, Yunnan 650032 Kunming, China; 2 Department of Orthopedics, The Second Xiangya Hospital of Central South University, 139# Middle Renmin Road, Changsha, Hunan 410011 China

**Keywords:** Developmental dysplasia of the hip, Periacetabular osteotomy, Double-incision approach, Learning curve

## Abstract

**Background:**

For periacetabular osteotomy, traditional approaches usually have a long learning curve. We aimed to evaluate the postoperative results and complications of periacetabular osteotomy under a new double-incision approach.

**Methods:**

The records of 58 consecutive patients (65 hips) who underwent periacetabular osteotomy using the new approach were retrospectively reviewed and evaluated. There were 52 women and 6 men with a mean age of 28.1 years at the time of surgery.

**Results:**

The average follow-up period was 35.2 months, during which no patients were converted to total hip arthroplasty. Complications included 6 hips (9.2%) with nerve dysesthesias and 1 hip (1.5%) with delayed wound healing. The mean operative time and intraoperative blood loss were 88.6 min and 402.8 ml, respectively. The mean modified Harris hip score had improved from 72.2 points preoperatively to 91.3 points at the last follow-up. Fifty-five patients (62 hips, 95.4%) were satisfied to their outcomes, and good preoperative functional score was associated with a satisfactory outcome. Furthermore, the average lateral center–edge angle, anterior center–edge angle and acetabular index angle were corrected well after surgery.

**Conclusion:**

Periacetabular osteotomy using modified Smith-Petersen or Bikini approach with posterolateral assisted small incision can be performed safely and with satisfactory results. In addition, this technique shortens the learning curve, and reduces the operating complexity, especially for beginner.

## Background

Acetabular dysplasia may lead to early development of joint disease [1, 2]. As yet, the exact mechanisms are not fully understood. Because the femoral head and acetabulum of hip dysplasia do not match well as a concentric circle, the abnormal stress concentration can lead to excessive stress in the local cartilage. This is a common cause of osteoarthritis that eventually requires total hip arthroplasty (THA) [3, 4]. In order to improve the matching rate between the femoral head and the acetabulum, prevent the occurrence of osteoarthritis or delay the further degeneration of articular cartilage, a variety of pelvic osteotomy have been designed to correct the abnormal acetabulum, including single innominate osteotomy, double osteotomy and triple osteotomy [5, 6]. The Bernese periacetabular osteotomy (PAO) described by Ganz et al. in the 1980s was a novel and commonly used surgical technique to reorient the acetabulum in adolescents and adults [1, 4, 7–10].

The PAO technique preserves the posterior column intact while preserving blood supply simultaneously, allowing for large rotation adjustments in multiple planes and maintaining pelvic continuity [6, 7]. Some studies have shown that the 10-and 20-year survival rate for the native hip joints after the Bernese PAO surgery are about 80–86% and 60%, retrospectively [11, 12]. However, there’s wide agreement that the Bernese osteotomy surgery is technically difficult with a long learning curve. In order to perform PAO safely with less complications, several surgical approaches (modified Smith-Petersen (S-P), ilio-inguinal, direct anterior, trans-trochanteric, and double approaches) have been tried over the years [4, 6]. Obviously, any such approaches should not compromise the adequacy of correction, functional outcome and complication rate. Some studies suggested that the modified S-P approach was the safest in terms of vessel and nerve protection and complication rate [6, 13, 14]. However, when the modified S-P approach was used for acetabular osteotomy, some studies have shown that the final function results are affected by inadequate visual filed exposure and soft tissue release [8, 15]. Because of the blindness and complexity of the anatomy, the PAO osteotomy with these approaches remains a major challenge, especially for beginner. The purpose of the present study was to report whether our double-incision approach was as safe and achieves the same morphological corrections as the single modified S-P or Bikini approach that quoted in the literature. In addition, we assessed the effect of possible preoperative risk factors on unsatisfactory outcomes after surgery.

## Methods

A retrospective study including patients underwent the Bernese PAO surgery was performed from February 1, 2016 to June 30, 2019. The study was approved by the Ethics Committee on Human Research of the 920th Hospital of Joint Logistics Support Force of Chinese People’s Liberation Army, and written informed consent was obtained from the participants.

**Fig. 1:**
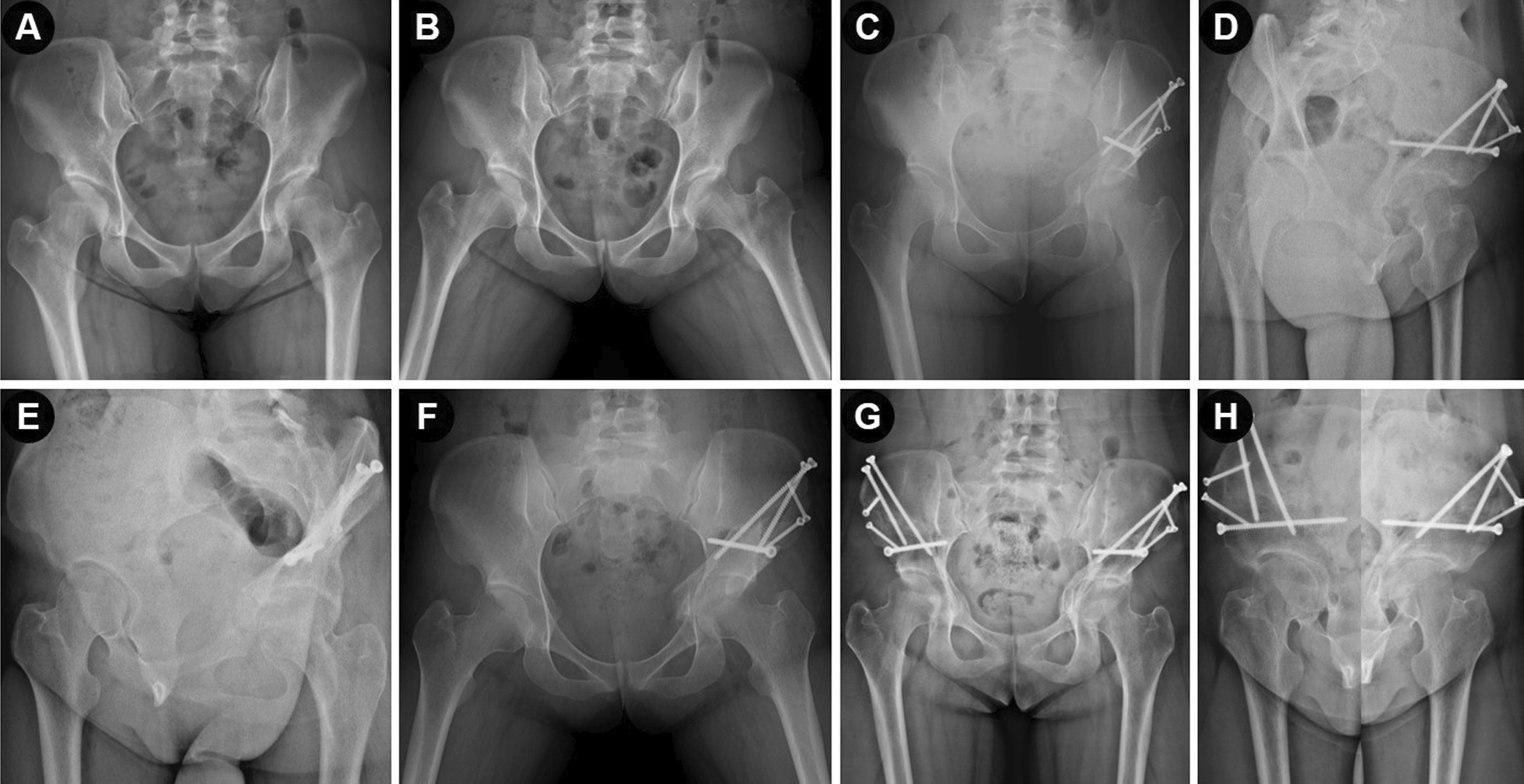
Preoperative radiograph and post-operative correction of a 22 years old woman with bilateral hip dysplasia. She was treated by Bernese PAO using the double-incision approach. **A**, **B** Preoperative radiographs of pelvis, showing bilateral acetabular dysplasia. **C**, **D** Postoperative anteroposterior pelvic radiograph and false-profile radiograph of the left hip at 2 months. **E**, **F** Preoperative radiographs of the right hip. **G**, **H** Postoperative anteroposterior pelvic radiograph and false-profile radiograph at 1 year (left hip) and 6 months (right hip)

**Fig. 2:**
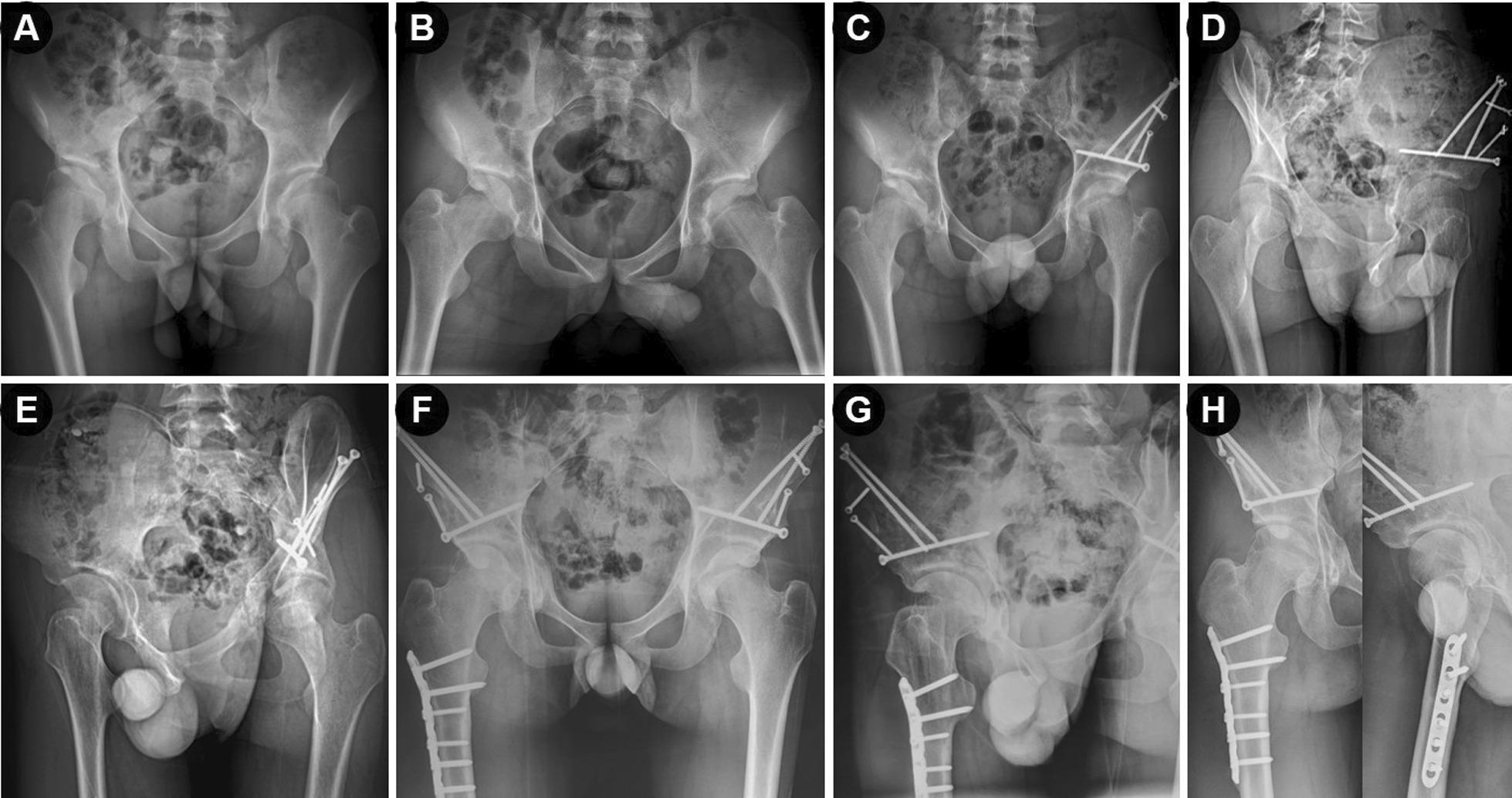
Preoperative radiographs and post-operative correction of an 18 years old man with bilateral hip dysplasia. He was treated by Bernese PAO and femoral derotation osteotomy under the double-incision approach. **A**, **B** Preoperative radiographs of the pelvis, showing bilateral acetabular dysplasia. **C**, **D** Postoperative anteroposterior pelvic radiograph and false-profile radiograph of the left hip at 3 months. **E** Preoperative false-profile radiographs of the right hip. **F**–**H** Postoperative radiographs at 1 year (left hip) and 9 months (right hip), showing good reconstruction of the hips

The Tönnis classification of hip osteoarthritis [16, 17] was used to grade osteoarthritis, and the Hartofilakidis classification system [18, 19] was used to assess the dysplastic hip. The indications for acetabulum osteotomy include symptomatic hip dysplasia that persists for more than 6 months after nonsurgical treatment failure, center–edge angle of Wiberg (LCE angle) < 25°, acetabular index (AI) > 10°, Tönnis grade 0–1, Y-type epiphyseal cartilage closure, and younger than 50 years of age [14, 20, 21]. Furthermore, the inclusion criteria included: (I) acetabular reorientation surgery with the use of Bernese PAO, (II) using the modified S-P or Bikini approach with posterolateral assisted incision. The exclusion criteria included: (I) patient who had undergone previous ipsilateral pelvic osteotomy or suffered from neuromuscular disease, (II) patient with osteo-chondroplasty, (III) patient with a history of pathological bone disease, and (IV) patient with incomplete medical records. The first 10 patients (10 hips) in our current study were excluded because these surgeries were performed with the help of other specialists. Finally, our present retrospective study involved 60 patients (67 hips) who met the inclusion criteria, among which 2 patients (2 hips) were lost to follow-up because of refusing to track on time. The clinical and radiographic data (Figs. 1 and 2) about the included patients were collected under the same criteria. Demographic characteristics of the included patients were noted from the medical records, and shown in Table 1.

**Table 1:** Demographics and clinical data for all patients

Variables	Mean ± SD (range)
No. of patients (hips)	58 (65)
Gender, n (%)	
Male	6 (10.3)
Female	52 (89.7)
Age (years)	28.1 ± 8.35 (11–49)
BMI (kg/m^2^)	22.0 ± 3.02 (17.4–30.4)
Side (hips, %)	
Left	30 (46.2)
Right	35 (53.8)
Preoperative Tönnis grade (hips, %)	
Grade 0–1	65 (100.0)
Hartofilakidis type (hips, %)	
Type Ι	65 (100.0)
Duration of surgery (min)	88.6 ± 17.91 (65–215)
Intraoperative blood loss (ml)	402.8 ± 87.28 (260–900)
Satisfaction of outcomes (hips, %)	
Satisfactory	62 (95.4)
Unsatisfactory	3 (4.6)
Follow-up (months)	35.2 ± 10.61 (18.0–56.0)

*SD* standard deviation, *BMI* body-mass index

### Surgical technique

**Fig. 3:**
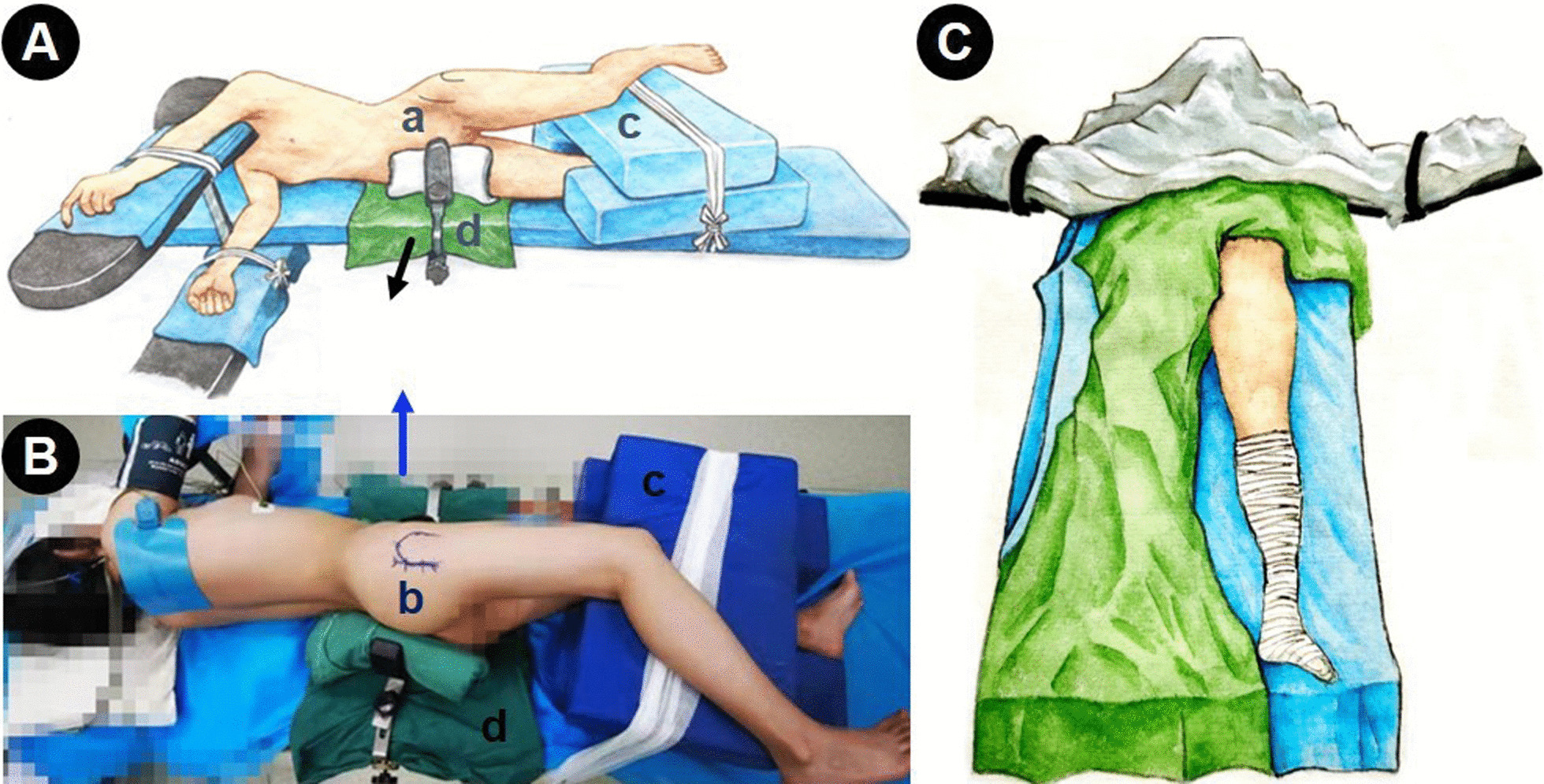
The intraoperative position change and surgical incision. **A**, **B** a new double-incision approach, including the modified S-P or Bikini approach (marked as “a”), one 5–8 cm assisted small incision on the rear edge of the greater trochanter (marked as “b”), three soft cushions (c) between the legs, play a role of protection and support, and placing a cloth pad (d) under the buttock in advance, which helps to directly pull (black arrow direction) and lift (blue arrow direction) during the operation to change the operation position. **C** After changed to the supine position, completing the pubic ramus and iliac bone osteotomy through modified S-P or Bikini incision

The surgical position is lateral position first and then supine position (Fig. 3), but only one operation skin disinfection and draping are required during the operation. Before transferring the patient from the trolley to the operating table, a 30–40 cm wide bed sheet was placed on the operating bed with the sheet under the patient’s buttocks for easy changing the position during operation. After induction of general anesthesia, the patient was placed on the operating bed in the lateral position with the pelvis lying over the radiolucent area and supported by the dam-boards (Fig. 3A, B). In order to obtain enough space for disinfection in the subsequent supine osteotomy area, the patient’s anterior support should not be higher than the pubic symphysis. In addition, to ensure adequate intraoperative hip disinfection, the disinfection range should reach at least the pubic symphysis in front, the upper edge to the iliac wings, and the posterior to the midline of the hip above the greater trochanter.

**Fig. 4:**
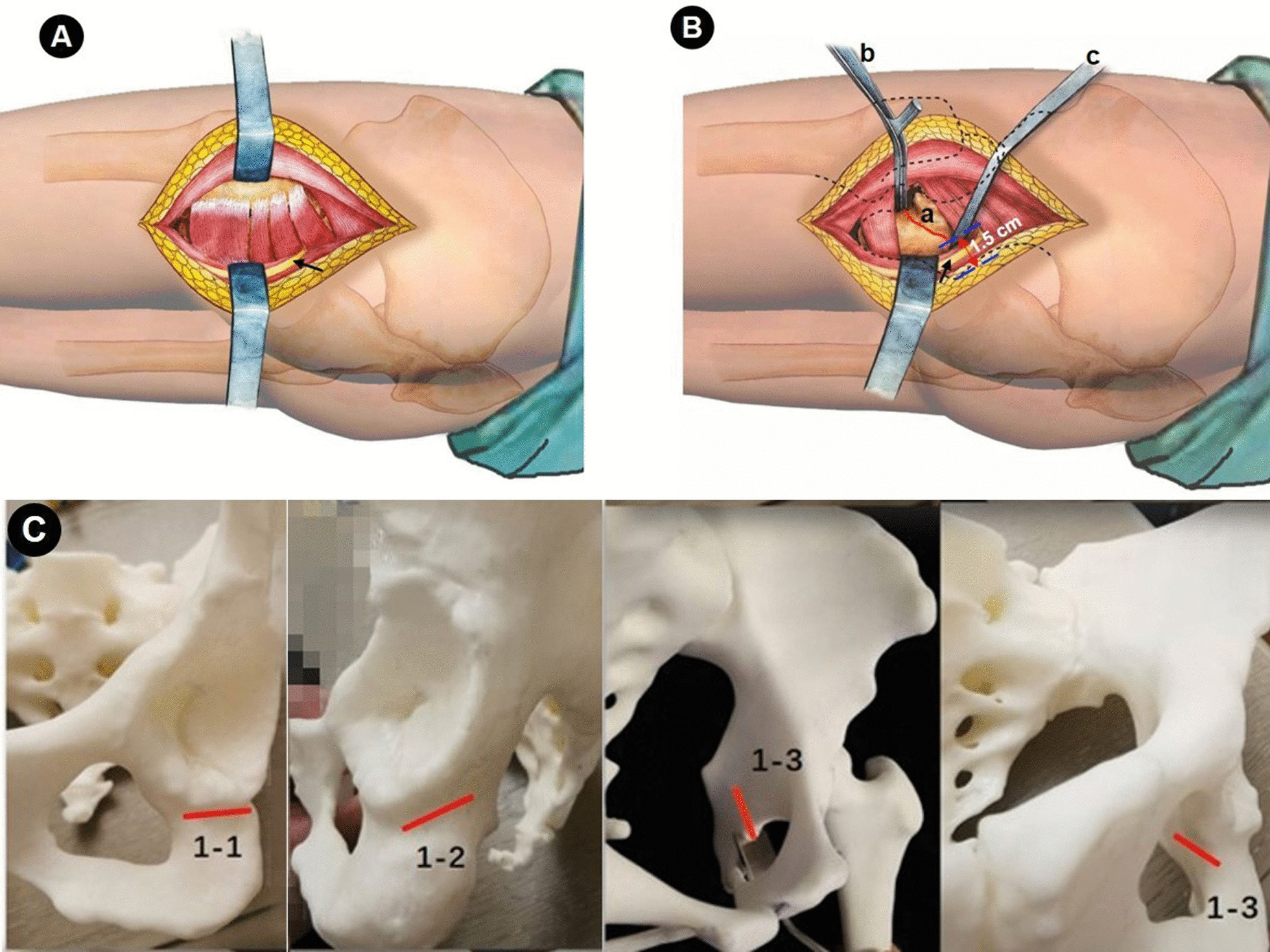
Schematic diagram of ischial osteotomy. **A** Expose the posterolateral assisted incision, and only the quadratus femoris, the inferior gemellus, and the obturator external muscles need to be exposed. **B** Enter into the intermuscular space of the upper margin of quadratus femoris, revealing the site of ischial branch osteotomy, that is the inferior acetabular sulcus (marked as “a”), then one Hoffman hooks (b) was placed into the obturator foramen along the front edge of the inferior acetabular sulcus, and another pointed Hoffman hooks (c) was hit into bone at 1.5 cm away from the edge of the posterior column, the sciatic nerve (black arrow) was protected by “S” hook and extending the affected lower limbs simultaneously. **C** 1–1, 1–2, and 1–3 represent the cortex of the inferior acetabular sulcus, lateral and medial cortex of the lower edge of the acetabulum, respectively

After flexion of the knee and internal rotation of the affected side to the lateral greater trochanter completely upward, a 5–8 cm incision was made that was behind and paralleled to the body surface of the great trochanter (Fig. 3B). After layer by layer incision of the skin, tensor fascia lata and the bursae associated with the greater trochanter of the femur, surgeon can see the attachment point of the quadratus femoris (Fig. 4A). In the lower margin space of obturator externus muscle, which lies on the upper edge of the quadratus femoris and about 1 cm behind the muscle attachment point, the blunt dissection with finger assistance was made to depths until the inferior acetabular sulcus is reached. During the exposure process, the sciatic nerve wrapped by the yellow fat layer can be seen, and it should be properly protected with an “S” retractor. Striping the periosteum forward and backward along the inferior acetabular groove with a narrow long periosteum detacher, and more attention should be payed to stretch the affected limb as far as possible when strip the periosteum backward. Then, using two Hoffman retractors to exposure the osteotomy area of ischium ramus (Fig. 4B). Subsequently, a 1.5 cm wide straight osteotome was used to perform the ischial osteotomy according to the marked line (Fig. 4B) and maintaining an angle of 10°–15° with the coccyx point. More attention should be payed to ensure that all three cortical bones are completely transect and that the posterior column remain 1–2 cm osseous continuity (Fig. 4C). In order to safely and completely resect the cortical bone of transverse groove of the acetabulum under a good view, the osteotome should be close to the anterior Hoffman retractor. When performing the upper posterior osteotomy along the marked line, the surgical assistant should pay attention to extend the affected lower limbs to relax the sciatic nerve. Normally, the osteotomy of the ischial branch can be done with an osteotome of this width for two times. When the ischial osteotomy is completed, the incision is sutured layer by layer.

**Fig. 5:**
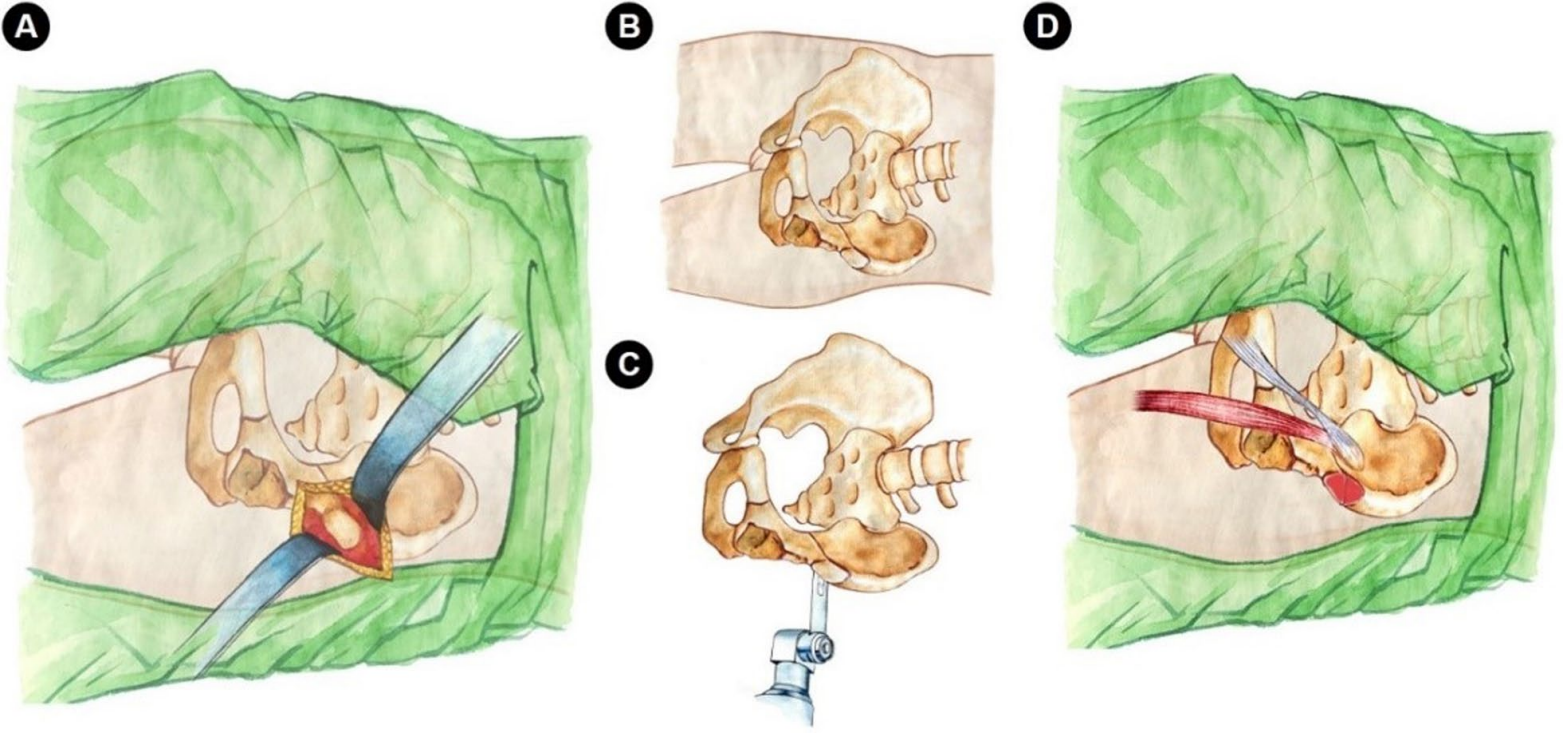
**A** Expose the attachment point of the anterior superior iliac spine through the modified S-P or Bikini approach. **B–D** Cut a 2–3 cm length anterior superior iliac spine block, and pull the sartorius muscle and the fulcrum toward the inside

**Fig. 6:**
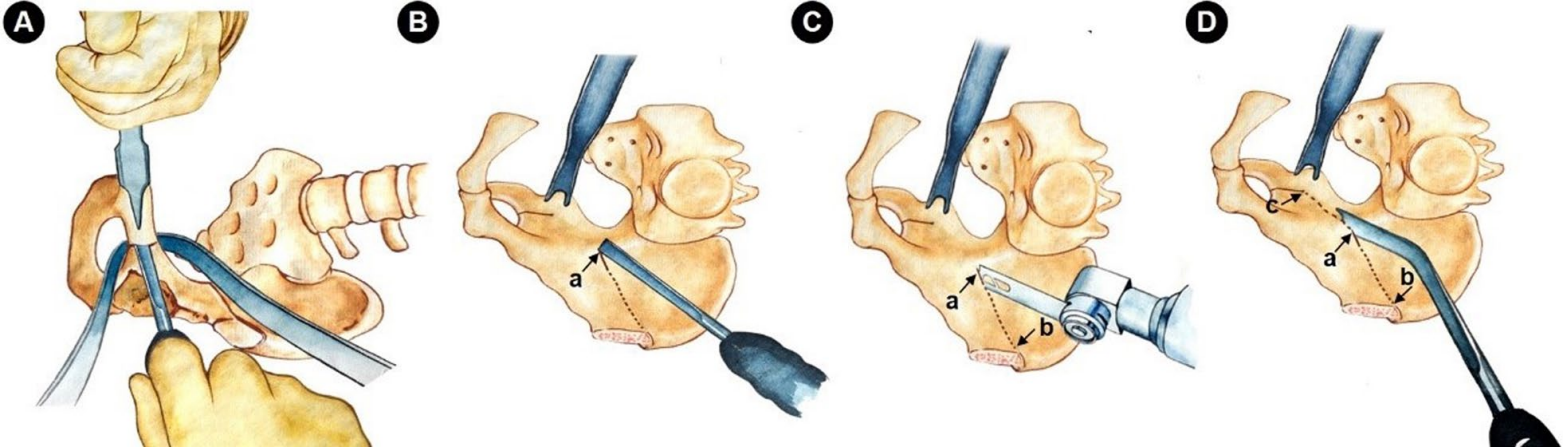
Schematic diagram of pubic and iliac osteotomy. **A** The pubic ramus osteotomy, two Hoffman hooks were inserted into the obturator under the periosteum to protect the osteotomy site. **B** Mark the point “a” (the solid line) with a 1.5 cm straight osteotome at the edge of the arcuate line about 4 cm from the anterior edge of the acetabulum. **C** Use a swing saw to cut the ilium along the dotted line at point “b” to the marked point “a”. **D** Use a double shoulder osteotome along the lower edge of the marked point “a”, 1–1.5 cm from the posterior column, and cut the bone from point “a” to point “c”

**Fig. 7:**
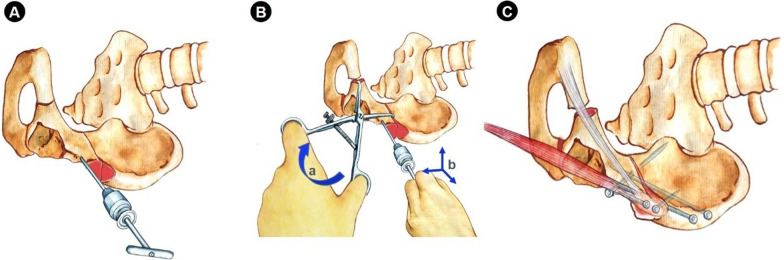
The rotation and fixation of acetabular fragments. **A** Place the Schanz nail between the upper and lower anterior superior iliac spine and hold it with a T handle. **B** The reduction towel clip was clamped at the pubic branch osteotomy end and the ilium osteotomy end respectively, so as to cooperate with the T handle to complete the rotation coverage, including rotate clockwise when looking down vertically (a), lifting, pressing down and turning inversion (b). **C** The fragments were fixed with stainless steel screws (Zimmer, 3.5-mm screw diameter and 2–5 cm length for fixation of anterior superior iliac spine, 4.5-mm screw diameter and 5–13 cm length for the others), triangular support and fixation as much as possible, and finally fix the bone block of sartorius muscle stop

Subsequently, with the help of itinerant nurse and anesthetist, the patient was changed from a lateral position to a supine position (Fig. 3C). The itinerant nurse removes the restraint belts of patient’s limbs and the anteroposterior dam-board firstly, then pulls the prepared sheet in parallel upwards to convert the position of patient. Due to the change in patient’s position, the relative contaminated area of the sterile sheet on the unaffected side would rise slightly. At this time, it is recommended to cover the sterile sheet on the unaffected side again. A traditional pelvic osteotomy surgery was then performed as described using a modified S-P or Bikini approach [6–8]. The initial skin incision is about 10 cm length, and the incision can be extended later depending on the surgical exposure (Fig. 5A). The skin and subcutaneous fascia were dissected and the lateral femoral cutaneous nerve (LFCN) was pulled inward without intentional separation, then an osteotomy of the anterior superior iliac spine was performed (Fig. 5B–D). The sartorius muscle attached to the anterior superior iliac spine was pulled inward along with the osteotomy block, gradually exposing the inner plate of the iliac crest and the pubic tuberosity. The iliopsoas tendon, femoral nerve and vessel bundle tract were protected and pulled inward, and the pubic was exposed by sub-periosteum dissection. Two Hoffman hooks were placed in the subpubic obturator foramen to expose and protect the pubis ramus for osteotomy, or the pubic could be osteotomy at about 1 cm inside the pubic tuberosity according to the perspective position (Fig. 6A). After exposing the ilium quadrilateral, ischial notch and ischial spine fully, the iliac and quadrilateral osteotomy were then performed, and this osteotomy line was connected with the first acetabular inferior sulcus osteotomy line (Fig. 6B–D). After confirming the complete periacetabular osteotomy, the acetabular fragments were rotated to obtain proper correction for a LCE angle from 25° to 35° and an AI from 0° to 5° (Fig. 7A, B), to get well covered of the femoral head which were confirmed by C-arm fluoroscopy, and to avoid the cross-over sign on the posterior and anterior X-ray [1, 8, 22]. After initial fixation of the bone fragments, the hip range of motion was measured and the acetabular osteotomy was finally fixed with three stainless steel screws (Fig. 7C). Move the affected lower limb again to check whether the fixation of fragment is stable. The osteotomy of the anterior superior iliac spine was reattached with the use of screw. After repeated flushing of the wound, subcutaneous tissue and skin were routinely sutured layer by layer.

Aiming to reduce blood loss and transfusion rates, we have administrated an intravenous infusion of tranexamic acid (TXA) (25 mg per 1 kg weight) before surgery. A second-generation cephalosporin was transfused at least 30 min before skin incision and continued less than 24 h after operation. Mechanical prophylaxis was routinely administrated against venous thromboembolism with the help of rehabilitation therapists. Crutch-walking with partial weight-bearing was instituted from 1 week postoperatively to 4 weeks, after that weight-bearing as tolerated was allowed.

### Clinical and radiographic assessment

Before operation, every month within 3 months after operation, 6 months and 1 year after operation, and then annually during the follow-up period, clinical and radiological evaluations were performed on all patients (Figs. 1 and 2). Clinical assessment was focus on patients’ demographics, previous management, daily activity and symptoms of acetabulum. The modified Harris hip scores (mHHS) [5] and visual analog scale (VAS) [1, 23] were applied and recorded for the clinical and functional evaluation. Trendelenburg sign was applied to assess abduction strength. Radiographic evaluation was based on the standing anteroposterior radiograph and false-profiles radiograph of pelvis [5]. Two independent authors blindly reviewed and assessed the radiology results to minimize bias and the inconsistent evaluation, if it occurs, was resolved through discussion and consensus with two other senior surgeons. In the present study, we graded the results according to the mHHS scores and Tönnis grade of the affected hip osteoarthritis [5]. We considered the result as satisfactory outcome if it was excellent or good result, and considered as unsatisfactory outcome if it was fair or poor result [5]. In addition, we have evaluated any possible significant risk factors associated with the unsatisfactory outcomes.

### Statistical analysis

The Wilcoxon signed-rank test or t-test was used to analyze difference between preoperative measurements and the last follow-up values of parameters. To analyze difference between the rates of satisfactory and unsatisfactory outcomes, we applied the nonparametric Wilcoxon rank-sum test for each continuous variable and applied Fisher exact test for each discrete variable. Values of *p* < 0.05 were considered to indicate statistical significance. All statistical analysis was performed using SPSS 19.0 software (SPSS Inc., Chicago, IL, USA).

## Results

The mean operative time and intraoperative blood loss were 88.6 (65 to 215) min and 402.8 (260 to 900) ml, respectively. No patients were converted to a THA at the last follow-up. There was no patient of sciatic nerve injury, or deep-vein thrombosis, or nonunion of the osteotomy site. At the time of last follow-up, there was no Trendelenburg-positive hip joint, and no patients experienced infection after osteotomy surgery. Two patients (2 hips, 3.1%) require a post-operative allogenic blood transfusion. Six patients (6 hips, 9.2%) had hypoesthesia in the LFCN distribution, and two patients (2 hips, 3.1%) had no remission of this symptom at the last follow-up. Delayed wound healing (over 2 weeks) was occurred in one patient (1 hip, 1.5%) of the anterior incision, and the wound was healed with the prolonged wound dressing. At the last follow-up, pain had decreased in all patients, and the mean VAS reduced from 5.2 points preoperatively to 1.37 points postoperatively (*p* = 0.000) (Table 2).

**Table 2:** Pre-operative and the last follow-up comparison

Variables	Pre-operative*	The last follow-up*	Z/T-value	*p*-value
Tönnis grade (%)			− 0.816	0.414
Grade 0	52 (80.0)	55 (84.6)		
Grade 1	13 (20.0)	9 (13.8)		
Grade 2	0 (0)	1 (1.5)		
mHHS	72.19 ± 8.87	91.33 ± 4.42	− 23.630	0.000
VAS	5.23 ± 0.86	1.37 ± 0.94	39.502	0.000
LCE angle (°)	4.26 ± 9.17	32.47 ± 3.09	− 26.102	0.000
ACE angle (°)	0.69 ± 8.32	31.31 ± 4.73	− 32.051	0.000
AI angle (°)	26.28 ± 7.45	3.89 ± 3.04	26.601	0.000

*The values are given as the mean and standard deviation

*mHHS* modified Harris Hip Score, *VAS* visual analog scale, *LCE* later center-edge, *ACE* anterior center-edge, *AI* Acetabular index

For all patients, the mHHS significantly improved from a mean preoperative value of 72.2 points to 91.3 points at the last follow-up, and mHHS was excellent in 40 (61.5%) hips, good in 24 (36.9%) hips, and fair in one (1.5%) hip. The degree of affected hip osteoarthrosis decreased in 4 (6.2%) hips, remain unchanged in 59 (90.8%) hips, and increase in 2 (3.1%) hips. According to mHHS score and the change of Tönnis grade, 40 (61.5%) hips had an excellent result, 22 (33.8%) hips had a good result, 3 (4.6%) hips had a fair result. Three patients (3 hips; 4.6%) had an unsatisfactory result, and 55 patients (62 hips; 95.4%) with a satisfactory result. The average postoperative lateral center-edge, anterior center-edge and anterior center-edge were improved significantly than that of preoperative (*p* = 0.000) (Table 2). Asymptotic grade-1 heterotopic ossifications were found in 2 patients (2 hips; 3.1%) who received no advanced treatment. Furthermore, a good preoperative functional score maybe associated with a satisfactory outcome (*p* = 0.075), and the possible risk factors that were analyzed to determine whether they were related to unsatisfactory result are list in Table 3.

**Table 3:** Possible risk factors related to an unsatisfactory outcome

Variables	Satisfactory outcome* (N = 62)	Unsatisfactory outcome* (N = 3)	*p*-value
Age (year)	27.7 ± 8.07	37.0 ± 10.82	0.472
BMI (kg/m^2^)	22.0 ± 2.98	23.4 ± 4.14	0.812
Degree of OA (Tönnis grade)	0.19 ± 0.40	0.33 ± 0.58	1.000
Duration of surgery (min)	106.8 ± 39.22	95.0 ± 5.00	0.898
Intraoperative blood loss (ml)	492.1 ± 138.58	500.0 ± 100.00	1.000
Duration of follow-up (months)	35.5 ± 10.68	30.0 ± 9.17	0.741
Preoperative mHHS (points)	72.8 ± 8.46	58.7 ± 6.94	0.075
Preoperative VAS (points)	5.2 ± 0.85	6.0 ± 1.00	0.964
Preoperative LCE angle (°)	3.9 ± 9.22	12.2 ± 1.78	0.065
Preoperative ACE angle (°)	0.3 ± 8.17	9.7 ± 7.22	0.224
Preoperative AI angle (°)	26.5 ± 7.54	22.4 ± 4.10	0.514

*The values are given as the mean and standard deviation

*BMI* body-mass index, *OA* osteoarthrosis, *mHHS* modified Harris Hip Score, *VAS* visual analog scale, *LCE* later center-edge, *ACE* anterior center-edge, *AI* Acetabular index

## Discussion

Developmental dysplasia of the hip (DDH) is a congenital developmental deformity that is more common in women, and most patients have clinical symptoms between 20 and 40 years old [24]. In this study, among the 58 patients with a mean age of 28.1 years at time of surgery, 52 (89.7%) cases were women. In order to prevent subluxation or dislocation of the hip at the early stage, patient should reduce joint load and avoid high-intensity activities, and even consider preventive osteotomy. In term of surgery, the current mainstream method is mainly reconstructive acetabular osteotomy by changing the direction of the acetabulum, including Bernese PAO and acetabular rotation osteotomy [4, 11]. These osteotomy surgeries can restore the optimal physiological position of the acetabulum, increase the coverage of the acetabulum on the femoral head, block or delay the pathological process of osteoarthritis, and avoid or delay the implementation of arthroplasty. However, reconstruction of the acetabular osteotomy has high technical requirements, large trauma and various complications [4, 11, 13, 14]. Therefore, it is necessary to explore new surgical method to reduce surgical trauma and surgical complications. In this study, we have described a new, safe and effective modified S-P or Bikini approach with a posterolateral assisted small incision for the Bernese PAO.

As is known to all, the open surgical technique and PAO internal fixation are the accepted treatment options for acetabular dysplasia [4, 11, 12]. Over the past year, several surgical procedures have been tried for PAO. Khan et al. [14] have described a new, safe and effective minimally invasive periacetabular osteotomy using a modified S-P approach, and their results demonstrated that good correction can be achieved, with satisfactory functional results and a low complication rate. Ko et al. [5] have reported a modification of the spherical acetabular osteotomy with use of a modified Ollier approach, and found this approach is more readily learned compared with other periacetabular osteotomies. Isaksen et al. [1] reported that periacetabular osteotomy through the anterior intrapelvic approach can be performed safely and with satisfactory results at medium-term follow-up. In our current study, all patients underwent osteotomy using the modified S-P approach with a posterolateral assisted incision to routinely protect the integrity of the long head and the retroflexion head of the rectus femoris. Protecting the integrity of the rectus femoris through a posterolateral assisted incision reduces intraoperative bleeding, reduces surgical time and hospital stay, and reduces surgical complications, including sciatic nerve injury. Our results demonstrated that this method can achieve good correction effect, satisfactory functional effect and low complication rate. In addition, the functional improvement and reorientation normalization were not significantly different from those reported in the literature [8, 14]. The Bernese osteotomy with this new approach usually minimizes soft tissue and nerve damage. Therefore, we have reason to believe that this double-incision approach is a better choice for Bernese PAO.

Some reports have shown that an additional 1 to 1.5 h of the operation time was required when performing PAO with the use of a double-incision approach [8, 25]. In this study, the posterolateral assisted incision provided a relatively increase surgical filed of vision and effectively matched the procedures associated with PAO. On the one hand, surgical dislocation of the hip can be attempted through the posterolateral assisted incision when necessary. This posterolateral assisted incision can fully expose the acetabulum and femoral head, preserve blood supply to the femoral head, provide a nearly 360° view of the femoral head and acetabulum, contribute to the diagnosis and treatment of joint capsule disease, and protect the sciatic nerve. On the other hand, it helps complete ischial osteotomy under direct vision, reduce the difficulty of ischial osteotomy, and fully protect the sciatic nerve at the same time. Therefore, our results showed that none of the osteotomy lines penetrated the hip joint. None of the posterior columns of the acetabulum were fractured and none of the sciatic nerves were damaged. Furthermore, the imaging indicators and the average mHHS were significantly improved at the last follow-up, and the operation time did not increase significantly compared with traditional PAO [4, 26], which would be attributed to complete the ischium osteotomy under direct vision and reduce the time of C-arm fluoroscopy. At the last follow-up, the patients’ hip pain symptoms improved significantly compared to that before osteotomy. Only two (3.1%) patients had an increase in the grade of osteoarthrosis, and no patients had avascular necrosis of the femoral head during the follow-up period, but the long-term results require further follow-up.

The risk of blood transfusion required for the Bernese PAO procedure is very low, and the amount of blood loss is related to the patient’s postoperative functional exercise. For acetabular osteotomy, the amount of blood loss reported was different in previous literatures. Some scholars reported that the average blood loss was as high as 2000 ml [27], and some other reports shown that the mean blood loss was 800 ml, 1400 ml, or 2092 ml using the modified S-P approach, or I–II approach, or ilioinguinal approach, respectively [26]. However, these articles did not explain whether the volume of blood loss was intraoperative blood loss or perioperative blood loss. The allogenic blood transfusion rate is 1.2% after a modified S-P approach [14]. In the current study, the intraoperative estimated blood loss was determined from data on the clinical charts as the blood collected from suctioning and the weight of the saturated sponges. The mean intraoperative blood loss was 402.8 ml, and 2 patients required a post-operative allogenic blood transfusion. There are several main reasons for the relative reduction in blood loss. Firstly, we calculated the amount of bleeding during surgery, but did not include postoperative wound drainage and hidden hemorrhage. Secondly, the posterolateral assisted incision contributes to complete ischial osteotomy under direct vision, and this surgical method saves the surgeon’s time to touch the ischium from the front and avoids the disadvantage of inconvenient hemostasis of the anterior approach. Thirdly, the posterolateral assisted incision shortens the length of the conventional incision, and naturally reduces the soft tissue injury of the conventional incision. Finally, we had adopted an intravenous infusion of TXA for reducing blood loss and transfusion rates [28]. However, surgeons should dynamically monitor the serum blood coagulation profiles during the perioperative period, avoiding complications occur.

In this study, we observed a less frequency (1.5%) of delayed wound healing in patients with the double-incision approach, and no patient had postoperative superficial as well as deep infections. The patient with delayed wound healing was healed with prolonged wound dressing. The result could be attributed to a sufficiently bridge of intact skin between the two incisions. We had left an intact skin bridge of at least 5 cm width between the two incisions. Any delay in wound healing may put the patient at risk of deep infection. Although the literature had shown that the rate of infection after pelvic osteotomies was relatively low, any methods that reduces the risk of wound healing problems should be used [6, 7, 25]. Furthermore, the analysis of possible risk factors has identified that unsatisfactory outcome may be negative influenced by the poor preoperative functional score. In principle, the earlier the osteotomy operation, the better the effect.

Injury to LFCN is a well-known complication after the PAO surgery. However, the incidences of LFCN injury were reported differently from different surgical approaches and different research reports. The incidences of complete or incomplete LFCN injury have been reported from 30 to 63% with different surgical approaches [1, 6], and a system review shown the rate of complete LFCN injury was 6.14% [4]. LFCN nerve injury has been reported to be around 30% after a modified S-P approach [14], about 10.5% after a modified Ollier approach [5], about 14% after an anterior intrapelvic approach [21], and about 6.7% [24] after the operative techniques initially described by Ganz et al. [7]. When the tension induced by traction or compression is applied to a nerve, both the extrinsic and intrinsic vascular supplies would affect the neural ischemia. In the present study, the rate of LFCN injury was 9.2% and the rate of complete LFCN injury was 3.1%. We believed the relatively reduced rate of LFCN injury was owned to the posterolateral assisted incision, which can reduce the tension of performing the ischial osteotomy from the anterior approach. However, the limited sample would affect the incidence. Whatever approaches adopted, keeping the nerve within the fascial sleeve of the tensor fascia lata can help to protect the nerve.

In addition, there is a learning curve for the Bernese PAO which requires considerable experience before master this pelvis surgery. Khan OH et al. [14] acknowledged that there will undoubtedly be a learning curve with the use of minimally invasive technique through a modified S-P approach and they have consistently used the approach in more than 250 further patients. For the anterior intrapelvic approach, Isaksen KF et al. [1] believed that a learning curve for the operative surgeons should also be taken into consideration. Therefore, a learning curve for the operating surgeons must be considered [1, 8]. We had used the posterolateral assisted incision for completing ischial osteotomy under direct view without the help of image intensifier, which naturally reduces the complexity of PAO surgery, especially for beginner. There is no significant difference in clinical and radiographic assessment (including mHHS, VAS, and reliable acetabular reorientation) in the current studies [4, 6, 8, 14]. Our results have shown that the posterolateral assisted incision with modified S-P approach was safe and allows optimal re-orientation of the acetabulum. Overall, periacetabular osteotomy using modified Smith-Petersen or Bikini approach with posterolateral assisted small incision can not only get satisfactory results, but also shorten the learning curve, especially for beginner.

We acknowledge several limitations to this study. First, the study was a retrospective design, no randomization, and a relatively small sample size of patients. With the learning curve and relatively low incidence, it was hard to obtain a larger number of patients. But further study with larger sample is required in our future research. Second, while there is undoubtedly a learning curve for PAO using this double-incision approach, the learning time is significantly shorter than other traditional approaches. Finally, we cannot predict the long-term failure rate because the relatively short to mid-term follow-up period also could affect the clinical and radiographic result assessment. Further researches with larger sample and longer follow-up are required to assess the results of the use of this double-incision approach for Bernese PAO.

## Conclusion

The modified S-P or Bikini approach with a posterolateral assisted small incision, by providing a direct view for ischial osteotomy and reduced soft tissue and nerves injury, allows safe accurate osteotomies in a reasonable operative time, with reduced blood loss and transfusion requirement, low complications, satisfied functional outcome and good radiological correction. More importantly, this new double-incision approach of PAO has an advantage of reduced learning curve, especially for beginner.

## Data Availability

The datasets used and/or analyzed during the current study are available from the corresponding author on reasonable request.

## Abbreviations

THA: Total hip arthroplasty
PAO: Peri-acetabular osteotomy
AI: Acetabular index
mHHS: Modified Harris hip scores
VAS: Visual analog scale
LFCN: Lateral femoral cutaneous nerve
DDH: Developmental dysplasia of the hip
TXA: Tranexamic acid
UCLA: University of California, Los Angeles
S-P: Smith-Petersen
